# The impacts of air pollution on daily hospitalizations for acute bronchitis in children: a perspective from the coastal city of Shantou, China

**DOI:** 10.3389/fpubh.2026.1796373

**Published:** 2026-05-08

**Authors:** Junduo Chen, Chuangxing Lin, Guangyu Lin, Xiaoying Cai

**Affiliations:** The Second Affiliated Hospital of Shantou University Medical College, Shantou, China

**Keywords:** acute bronchitis, air pollution, children, hospital admissions, susceptible populations

## Abstract

**Background:**

The purpose of this study was to explore the impacts of ambient air pollution on daily hospitalizations for acute bronchitis in children from a coastal city perspective in China, and to further investigate the related susceptible populations.

**Methods:**

Daily hospitalizations for acute bronchitis in children from 2015 to 2019, as well as meteorological factors and air pollutants for the same period in shantou were collected. We applied a Poisson generalized additive model (GAM) to investigate the impacts of air pollutants (PM_2.5_, PM_10_, SO_2_, NO_2_, O_3_) on daily hospitalizations for acute bronchitis, with stratified analysis by sex, age and season.

**Results:**

A total of 11,228 hospitalizations for acute bronchitis in children were collected. The results showed that daily hospitalizations for acute bronchitis in children were significantly associated with SO_2_ and NO_2_, but not with PM_2.5_, PM_10_ or O_3_. Specifically, for every 10 μg/m^3^ increase in the concentration of SO_2_ and NO_2_ at lag07, the relative risk (RR) and 95% confidence interval (CI) for hospitalizations due to acute bronchitis in children were 1.2485 (1.1116–1.4023) and 1.1815 (1.1082–1.2596) respectively. Stratified analysis indicated that these associations were more pronounced in the boys, 0–2 years group and during the cold season.

**Conclusion:**

Short-term exposure to SO_2_ and NO_2_ significantly increased the risk of hospitalizations for acute bronchitis in children, particularly in boys, children aged 0–2 and during the cold season (November to March). Relevant government departments should take further preventive measures to effectively reduce air pollutant concentrations.

## Introduction

Acute bronchitis is a common respiratory disease caused by inflammation of the bronchial mucosa, mostly seen in children (1), with typical clinical symptoms including coughing, sputum production, fever, and wheezing (2, 3). The disease is somewhat self-limiting, but if acute bronchitis is not treated promptly, it may progress to asthma or chronic bronchitis (3, 4). For patients with underlying chronic heart and lung diseases, there is a high likelihood of severe hypoxia or ventilation deficiency, which seriously affects the patient’s physical and mental health as well as normal daily life (4, 5). Currently, the treatment of acute bronchitis often involves the use of antibiotics on top of general and supportive therapy, which not only increases the financial burden on patients but may also lead to antibiotic resistance, disruption of microbial balance, and adverse drug reactions, resulting in prolonged illness and recurrent episodes in some patients (6, 7). Therefore, exploring and analysing the risk factors for acute bronchitis can not only alleviate the serious disease burden on patients but also provide a scientific and reasonable basis for the effective prevention of acute bronchitis.

The onset of bronchitis in children is mainly related to factors such as viral or bacterial infections, allergic constitution, and low immunity. Common triggers include invasion by pathogens such as respiratory syntactical virus and influenza virus, as well as stimulation by cold air, dust, or second-hand smoke (3, 4, 5). In addition, studies have shown that air pollutants may also trigger lower respiratory tract diseases such as acute bronchitis. Fine particulate matter can not only deposit in the upper respiratory tract of the human body but also enter the lower respiratory tract, causing inflammatory reactions (8, 9). Gaseous pollutants such as SO_2_, NO_2_ and O_3_ can enter the body through respiration, potentially damaging the barrier function of the respiratory system, exacerbating inflammatory responses, and increasing airway reactivity (9, 10). A study conducted in Hefei, China, found that short-term exposure to traffic-related pollutants such as PM_2.5_, NO_2_ and CO can significantly increase the number of outpatient visits for acute bronchitis in children (11). Another study in Hong Kong, China indicated that exposure to both low and high concentrations of NO_2_ and SO_2_ can significantly increase the risk of hospitalization for acute bronchitis in children, whereas the effects of PM_10_ and O_3_ were not significant (12). Other epidemiological studies in the United States (13), South Korea (14) and European (15, 16) have shown that PM_2.5_ or PM_10_ is significantly positively associated with the onset of lower respiratory tract diseases in children, but some studies have not found a correlation between the two (17, 18). It can be seen that in studies conducted in different regions, the harmful effects of various air pollutants on the incidence of acute bronchitis vary in magnitude and intensity, showing a distinct regional difference. This situation may be related to various factors such as the level of air pollution in different regions, the types of pollutants, climate conditions, geographical features, traffic conditions, and population composition. However, existing studies provide very scarce evidence on the association between air pollution and acute bronchitis in children from the perspective of coastal cities in southern China.

This study collected daily hospitalizations for acute bronchitis in children, air pollutants and meteorological factors in the coastal city of Shantou from 2015 to 2019, aiming to explore the acute effects of air pollution on daily hospitalizations for acute bronchitis in children and to identify the corresponding vulnerable groups.

## Materials and methods

### Study area

Shantou is located on the eastern coast of Guangdong Province and is one of the earliest Special Economic Zones in China. It administers six districts (Chaonan, Chaoyang, Haojiang, Jinping, Longhu and Chenghai) and one county (Nanao), covering a total area of 2,199 square kilometers. By the end of 2024, Shantou had a resident population of 5.5755 million and achieved a regional gross domestic product of 316.797 billion yuan. Shantou has a South Subtropical Maritime Monsoon climate, with a generally mild and humid climate throughout the year.

### Data collection

Daily hospitalizations for acute bronchitis in children from 1 January 2015 to 31 December 2019 was obtained from the medical record information systems of three large tertiary hospitals in Shantou, including the First Affiliated Hospital of Shantou University Medical College, Chaonan Minsheng Hospital of Shantou, and the Second Affiliated Hospital of Shantou University Medical College. These three hospitals are the highest-level medical institutions in Shantou, with standardized diagnostic and treatment procedures. Each case of acute bronchitis is repeatedly reviewed and confirmed by experienced clinicians and professional technicians, which can better reflect the temporal trend of hospitalizations for children with acute bronchitis. The patient information we have collected includes admission date, sex, age and disease diagnosis. Patients with acute bronchitis included in this study were required to meet the following criteria: (a) Patients must reside in Shantou, with cases whose residence is outside Shantou or with incomplete residential information excluded; (b) Diagnoses must comply with the International Classification of Diseases, 10th Edition code: J20; (c) Patients readmitted within 30 days were excluded (19, 20); (d) Studies have shown that the proportion of children over 14 years old developing acute bronchitis is very small (11), so this study only included children aged 0–14 years old.

The daily 24-h mean concentration of air pollutants (PM_2.5_, PM_10_, SO_2_, and NO_2_) and the maximum 8-h average concentrations of O_3_ were obtained from the China Air Quality Real-time Monitoring Platform.^1^ To avoid the confounding effects of meteorological factors, we also collected daily average temperature (°C) and relative humidity (%) during the study period from the China Meteorological Science Data Sharing Service Network.^2^

### Statistical analysis

Spearman correlation is used to analyse the correlation between air pollutants and meteorological indicators. A correlation coefficient close to 1 indicates a stronger correlation between the two indicators, and two indicators with a correlation coefficient greater than 0.7 are not included in the same pollutant model.

Then, we summarized daily hospitalizations for children with acute bronchitis, air pollutants and meteorological data by date to establish a time series database for subsequent analysis. The daily acute bronchitis hospitalizations in children usually follows an over-dispersed Poisson distribution (deviance/df = 2.11), therefore this study used a quasi-Poisson generalized additive models (GAM) to investigate the impacts of air pollutants on acute bronchitis (21). This study determined the final model using the following methods: (1) adjusting long-term and seasonal trends in calendar time with a natural cubic spline function of seven degrees of freedom (*df*) per year (22, 23); (2) selecting a natural cubic spline function with three *df* to control for the potential confounding effects of meteorological factors (23, 24); (3) additionally adjusting for day-of-week (*DOW*) and holiday effects as dummy variables. The model is shown as follows (Equation 1):


$$\begin{array}{l} \log\;E(Y_{t})=\beta P_{t}+\mathrm{ns}(\text{Temperature},3)+\mathrm{ns}(\begin{array}{l} \text{Relative}\;\\ \text{humidity},3 \end{array})+\\ \text{Holiday}+\mathrm{DOW}+\mathrm{ns}(\text{time},7\times 5)+\text{intercept} \end{array}$$ (1)

In the above model, *β* is the log-relative rate of the exposure-response relation between air pollutants and acute bronchitis, P*_t_* is the concentration of air pollutants, *t* is the observation day, and E (Yt) is the expected number of daily hospitalizations for acute bronchitis on day *t*.

We used the following methods to test the robustness of the model: First, consistent with several previously published studies (24, 25), we explored the lag patterns of the associations using single-day lag models (lag 0 to lag 7) and moving average exposure of multiple days (lag01 to lag07). Second, after adjusting for other co-pollutants, we constructed a two-pollutant model. Third, the degrees of freedom for the time trend were adjusted to 6–9, and those for average temperature and relative humidity were adjusted to 3–5.

In addition, we conducted subgroup analyses by sex (boys and girls), age (<3 years, 3–6 years and 7–14 years) (25), and season (warm season and cold season). The inter-group heterogeneity test was performed using the following formula (Equation 2):


$$(Q1-Q2)\pm 1.96\;\sqrt{(SE_{1})2+(SE_{2})2}$$ (2)

In the formula, Q1 and Q2 are the estimates values of the subgroups, and SE_1_ and SE_2_ represent their standard errors, respectively (26). In this study, the results are expressed as the relative risk (RR) and 95% confidence interval (CI) of daily hospitalizations for acute bronchitis in children associated with per 10 μg/m^3^ increase in pollutant concentrations.

The statistical analyses involved in this study were performed using R software (version 4.0.2), and model fitting was done using the “mgcv” package. *p* < 0.05 was considered statistically significant.

## Results

### Descriptive statistics

**Figure 1:**
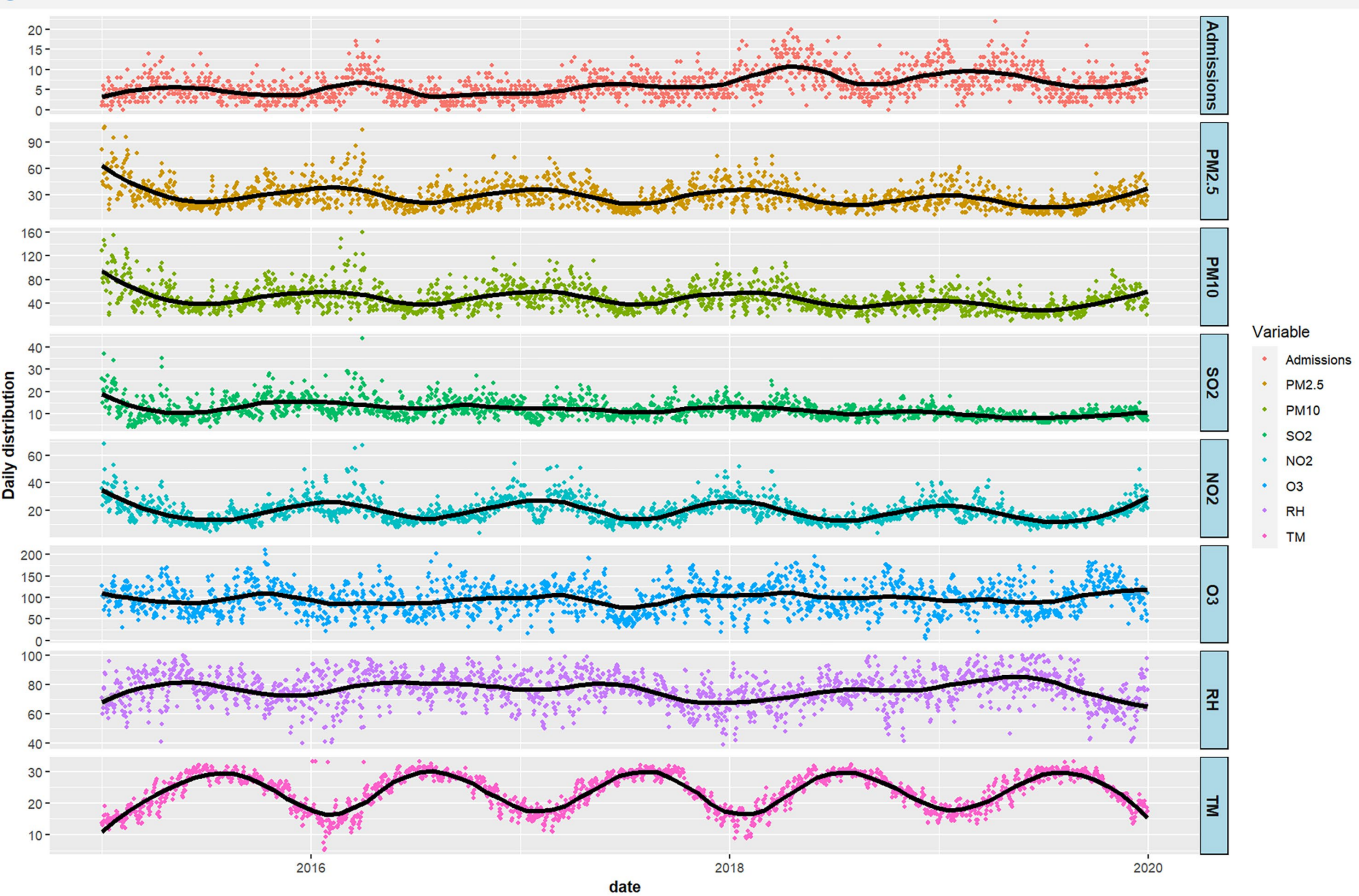
Time series distributions of daily acute bronchitis admissions, meteorological variables and air pollutants in Shantou, China, 2015–2019.

Descriptive statistics of daily hospitalizations for acute bronchitis in children, meteorological factors and air pollution variables from 2015 to 2019 are shown in Table 1. We identified a total of 11,228 pediatric patients with acute bronchitis. The number of hospitalized boys and girls with acute bronchitis was similar (boys, 5,686; girls, 5,542). In terms of age, children under 3 years accounted for 48.6% (5,459 cases), children aged 3–6 years accounted for 15.4% (1731 cases), and children aged 7–14 years accounted for 36% (4,038 cases). The number of hospitalizations in the cold season was slightly higher than that in the warm season (4,676 vs. 4,425). During the study period, the daily average concentrations of air pollutants were 28.1 μg/m^3^ for PM_2.5_, 11.8 μg/m^3^ for SO_2_, 19.7 μg/m^3^ for NO_2_, 47.6 μg/m^3^ for PM_10_, and 97.1 μg/m^3^ for O_3_. The daily average temperature and relative humidity during the same period were 23.6 °C and 76.6%, respectively. Time series distributions of daily hospitalizations for acute bronchitis in children, meteorological factors, and air pollutants are shown in Figure 1. It can be seen that the concentrations of PM_2.5_, PM_10_, and NO_2_ peak in winter, while O_3_ levels are higher in summer; SO_2_ shows a decreasing trend year by year, while daily hospitalizations for acute bronchitis shows an increasing trend over the years.

**Table 1:** Basic description of daily acute bronchitis hospitalizations, meteorological variables and air pollutants in Shantou, China, 2015–2019.

Variables	Sum	Mean	SD	Minimum	P10	P25	P50	P75	P90	Maximum
Acute bronchitis	11,228	6.15	3.50	0	2	4	6	8	11	22
Boys	5,686	3.11	2.81	0	0	1	2	5	7	16
Girls	5,542	3.04	1.78	0	0	2	3	4	5	11
0–2 years old	5,459	2.98	2.81	0	0	1	2	4	7	17
3–6 years old	1,731	0.95	0.96	0	0	0	1	2	2	4
7–14 years old	4,038	2.21	1.58	0	0	1	2	3	4	10
Warm season	4,425	2.42	3.51	0	0	0	0	5	8	19
Cold season	4,676	2.56	3.78	0	0	0	0	5	8	17
PM_2.5_ (μg/m^3^)	—	28.07	14.15	6	13	18	25	35.7	47	108
PM_10_ (μg/m^3^)	—	47.59	20.52	10	25	33	44	58	74	159
NO_2_ (μg/m^3^)	—	19.69	8.22	4	11	14	18	24	30	68
SO_2_ (μg/m^3^)	—	11.81	4.21	4	7	9	11	14	17	44
O_3_ (μg/m^3^)	—	97.18	34.17	4	54	70	98	120	143	211
Mean temperature (°C)	—	23.55	5.67	5.1	15.5	18.8	24.5	28.5	30.2	33.2
Relative humidity (%)	—	76.63	11.12	39.3	61.8	69.8	77.0	84.8	90.1	100.0

SD, standard deviation; P_10_, P_25_, P_50_, P_75_, P_90_: the10th, 25th, 50th, 75th, 90th percentile; Warm season, May to September; Cold season, November to March; PM_2.5_, particulate matter with aerodynamic diameter less than 2.5 μm; PM_10_, particulate matter with aerodynamic diameter less than 10 μm; NO_2_, nitrogen dioxide; SO_2_: Sulfur dioxide; O_3_, Ozone.

Supplementary Table S1 displays the Spearman’s correlation coefficients between meteorological factors and air pollutants in Shantou, China. There is a strong positive correlation between PM_2.5_ and PM_10_ (0.94, *p* < 0.001), a weak correlation between SO_2_ and NO_2_ with O_3_, while other pollutants show a moderate positive correlation with each other. In contrast, air pollutants are negatively correlated with meteorological variables.

### Results of model fitting

We used a single-pollutant model to determine the impacts of air pollutants on hospitalizations for acute bronchitis. Table 2 displays the RRs and 95% CIs of daily hospitalizations for acute bronchitis at specific lag days (0–7) and moving average lag days (01–07) associated with a 10 μg/m^3^ increase in air pollutants in single-pollutant models. We found that short-term exposure to SO_2_ and NO_2_ was significantly associated with an increased risk of daily hospitalizations for acute bronchitis. For every 10 μg/m^3^ increase in pollutant concentrations, the significant single-day adverse effect of SO_2_ lasts from lag1 (RR = 1.0752, 95% CI: 1.0116–1.1426) to lag4 (RR = 1.1235, 95% CI: 1.0587–1.1923), with multi-day moving average lag days persisting from lag01 (RR = 1.0960, 95%CI: 1.0186–1.1793) to lag07 (RR = 1.2485, 95% CI: 1.1116–1.4023), reaching the maximum effect at lag05 (RR = 1.2561, 95% CI: 1.1343–1.3908). For NO_2_, the significant single-day adverse effect lasts from the day of exposure (RR = 1.0596, 95% CI: 1.0239–1.0965) to lag04 (RR = 1.0623, 95%CI: 1.0275–1.0984), with multi-day moving average lag days persisting from lag01 (RR = 1.0807, 95% CI: 1.0381–1.1250) to lag07 (RR = 1.1815, 95% CI: 1.1082–1.2596). However, no significant association was found between PM_2.5_, PM_10_, or O_3_ and hospitalizations for acute bronchitis.

**Table 2:** RR and 95% CI of daily acute bronchitis hospitalizations in children with per 10 μg/m^3^ increase in pollutants at different lag days.

Lag	PM_2.5_	PM_10_	SO_2_	NO_2_	O_3_
0	1.0107 (0.9920–1.0298)	1.0082 (0.9947–1.0219)	1.0599 (0.9947–1.1294)	1.0596 (1.0239–1.0965)*	0.9984 (0.9904–1.0065)
1	1.0110 (0.9926–1.0298)	1.0096 (0.9969–1.0224)	1.0752 (1.0116–1.1426)*	1.0563 (1.0208–1.0931)*	1.0007 (0.9936–1.0080)
2	1.0102 (0.9920–1.0287)	1.0076 (0.9953–1.0201)	1.0778 (1.0153–1.1442)*	1.0645 (1.0295–1.1006)*	1.0062 (0.9995–1.0129)
3	1.0159 (0.9978–1.0343)	1.0112 (0.9990–1.0236)	1.1047 (1.0408–1.1726)*	1.0645 (1.0297–1.1004)*	1.0035 (0.9969–1.0101)
4	1.0121 (0.9941–1.0303)	1.0109 (0.9988–1.0233)	1.1235 (1.0587–1.1923)*	1.0623 (1.0275–1.0984)*	1.0049 (0.9983–1.0114)
5	0.9981 (0.9807–1.0159)	1.0014 (0.9895–1.0134)	1.0537 (0.9927–1.1184)	1.0251 (0.9911–1.0602)	1.0035 (0.9970–1.0101)
6	0.9976 (0.9803–1.0152)	1.0013 (0.9895–1.0132)	0.9903 (0.9325–1.0516)	1.0154 (0.9816–1.0503)	1.0043 (0.9978–1.0108)
7	0.9980 (0.9807–1.0156)	0.9974 (0.9857–1.0093)	1.0167 (0.9578–1.0793)	1.0342 (0.9998–1.0697)	1.0035 (0.9971–1.0099)
01	1.0149 (0.9932–1.0371)	1.0122 (0.9970–1.0276)	1.0960 (1.0186–1.1793)*	1.0807 (1.0381–1.1250)*	0.9996 (0.9908–1.0086)
02	1.0188 (0.9943–1.0439)	1.0145 (0.9978–1.0314)	1.1280 (1.0401–1.2233)*	1.1085 (1.0596–1.1597)*	1.0037 (0.9944–1.0131)
03	1.0263 (0.9992–1.0542)	1.0188 (0.9996–1.0380)	1.1769 (1.0776–1.2854)*	1.1359 (1.0813–1.1934)*	1.0050 (0.9953–1.0149)
04	1.0313 (0.9998–1.0601)	1.0229 (0.9997–1.0437)	1.2344 (1.1226–1.3573)*	1.1603 (1.1002–1.2236)*	1.0069 (0.9966–1.0174)
05	1.0292 (0.9974–1.0619)	1.0225 (0.9999–1.0447)	1.2561 (1.1343–1.3908)*	1.1657 (1.1013–1.2340)*	1.0081 (0.9972–1.0192)
06	1.0271 (0.9934–1.0618)	1.0224 (0.9998–1.0459)	1.2421 (1.1138–1.3852)*	1.1676 (1.0990–1.2404)*	1.0095 (0.9981–1.0212)
07	1.0256 (0.9901–1.0624)	1.0206 (0.9975–1.0443)	1.2485 (1.1116–1.4023)*	1.1815 (1.1082–1.2596)*	1.0107 (0.9986–1.0229)

*p < 0.05.

### Stratified analysis

**Figure 2:**
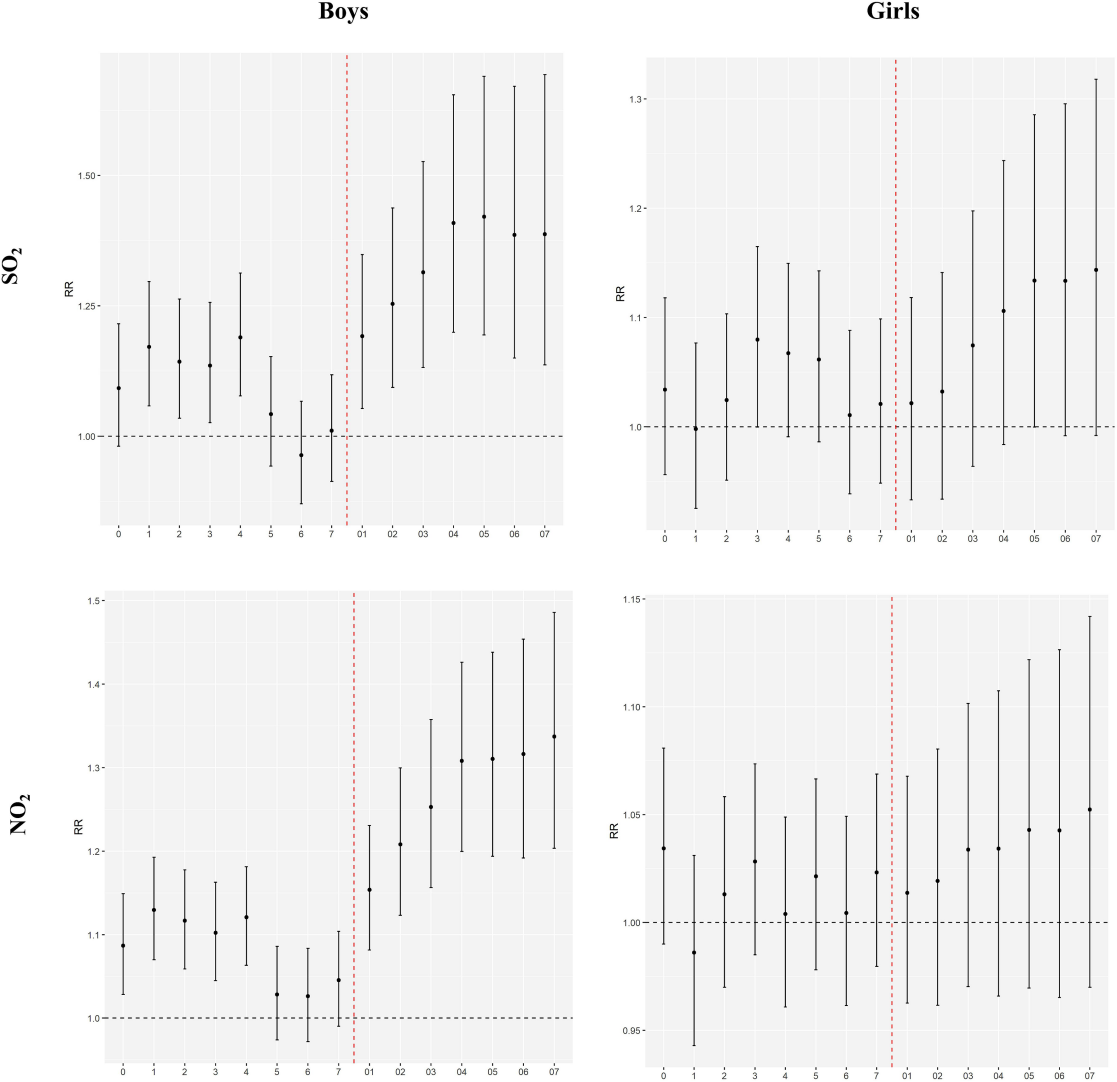
Effects of air pollutants on hospital admissions for acute bronchitis among different sex groups in Shantou, China, 2015–2019.

**Figure 3:**
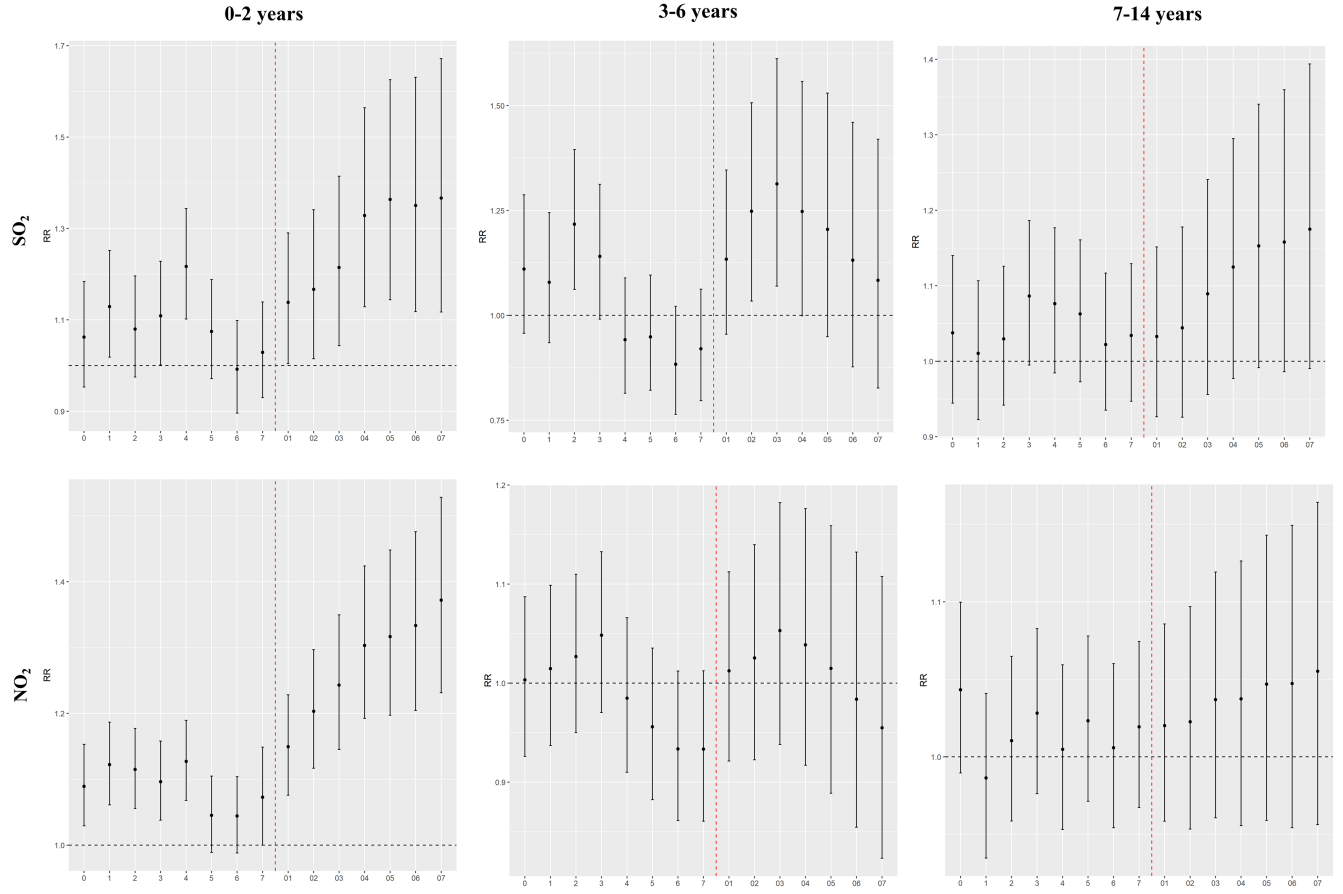
Effects of air pollutants on hospital admissions for acute bronchitis among different age groups in Shantou, China, 2015–2019.

Figures 2, 3 show the results of sex- and age-specific analyses. We found that boys and children aged 0–2 years seem to be more susceptible to air pollutants than their corresponding groups (Supplementary Tables S2–S6). In the boys’ group, for every 10 μg/m^3^ increase in SO_2_ and NO_2_ concentrations at lag07, the associated RRs were 1.3875 (1.1367–1.6936) and 1.3372 (1.2035–1.4859), respectively. In the 0–2 years age group, for every 10 μg/m^3^ increase in SO_2_ and NO_2_ concentrations at lag07, the associated RRs were 1.3667 (1.1173–1.6718) and 1.3737 (1.2314–1.5281), respectively.

**Figure 4:**
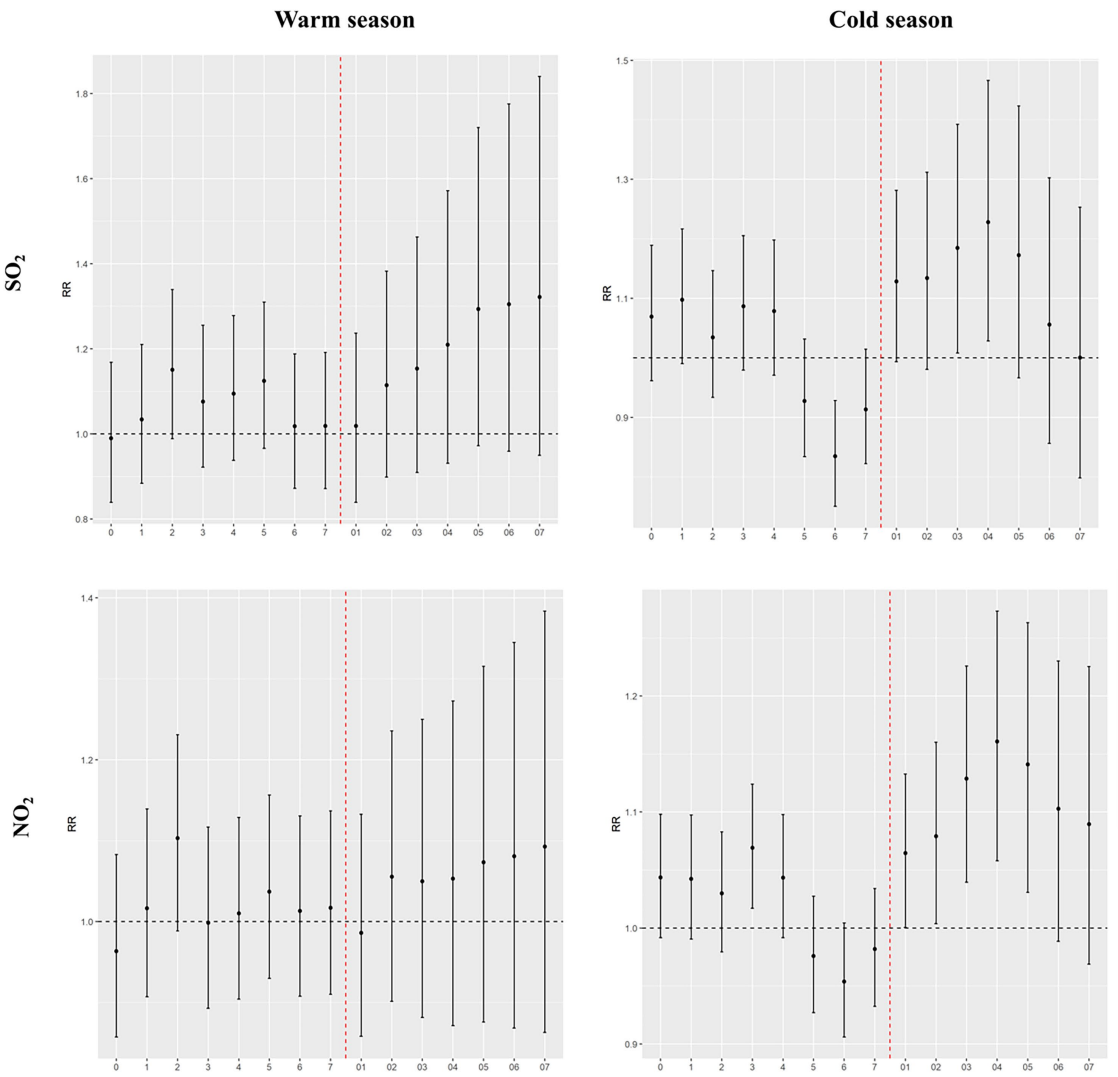
Effects of air pollutants on hospital admissions for acute bronchitis among different season groups in Shantou, China, 2015–2019.

The results of the seasonal-specific analysis are shown in Figure 4. We found that the significant adverse effects of SO_2_ and NO_2_ were stronger in the cold season than in the warm season (Supplementary Tables S7, S8). In the cold season group, for every 10 μg/m^3^ increase in SO_2_ and NO_2_ concentrations at lag04, the corresponding RRs were 1.2280 (1.0284–1.4663) and 1.1607 (1.0580–1.2733), respectively. However, no significant adverse effects of air pollutants were observed during the warm season.

### Sensitivity analyses

Table 3 shows the RR and 95% CI for daily hospitalizations for acute bronchitis associated with a 10 μg/m^3^ increase in the concentrations of SO_2_ and NO_2_ in two-pollutant models. It can be seen that regardless of adding any other air pollutant to the SO_2_ or NO_2_ model, its effect value shows a slight increase or decrease, but still remains statistically significant. In addition, after adjusting the *dfs* for time trend (6–9) or for daily average temperature and relative humidity (3–5), the effect estimates and confidence intervals slightly increased or decreased, but the results remained statistically significant (Supplementary Tables S9, S10).

**Table 3:** RR and 95% CI for daily acute bronchitis hospitalizations in children associated with a 10 μg/m^3^ increase in the concentrations of SO_2_ and NO_2_ in two-pollutant models.

Model	RR	95% CI
SO_2_		
Single pollutant model	1.2561	1.1343–1.3908^*^
+PM_2.5_	1.3101	1.1519–1.4901^*^
+PM_10_	1.2964	1.1371–1.4779^*^
+NO_2_	1.0646	1.0191–1.1330^*^
+CO	1.0225	1.0116–1.0336^*^
+O_3_	1.2536	1.1259–1.3957^*^
NO_2_		
Single pollutant model	1.1815	1.1082–1.2596^*^
+PM_2.5_	1.2065	1.1215–1.2979^*^
+PM_10_	1.1925	1.1102–1.2809^*^
+SO_2_	1.1738	1.0722–1.2852^*^
+CO	1.0224	1.0144–1.0306^*^
+O_3_	1.1755	1.1014–1.2548^*^

The most significant effects with multi-day lag (lag05 for SO_2_ and lag07 for NO_2_) were used. *p < 0.05.

## Discussion

This study investigated the association between air pollution and daily hospitalizations for acute bronchitis in children in Shantou, a subtropical marine climate city, and identified potentially susceptible populations. The results showed that daily hospitalizations for acute bronchitis in children was significantly associated with SO_2_ and NO_2_, but not with PM_2.5_, PM_10_ or O_3_. Boys and children aged 0–2 were more susceptible to SO_2_ and NO_2_. In addition, the effects of air pollutants were more pronounced in the cold season. Our findings further enrich the evidence of the association between air pollution and hospitalizations for acute bronchitis in China.

In recent years, with the gradual improvement of people’s health awareness and the increasing attention paid to the health risks caused by air pollution, studies on the association between air pollutants and the incidence of respiratory diseases have been increasing. Among these, a relatively large number of studies have focused on particulate matter (such as PM_2.5_ and PM_10_), but the conclusions drawn from different studies are inconsistent. For example, a study conducted in Guangzhou, China, found that different PM_2.5_ exposure indicators (daily mean, daily excessive concentration hours, and hourly peak) all significantly increased the outpatient visits for acute lower respiratory infections in children (27). A time-series study across multiple cities in Sichuan Province, China, showed that for every 10 μg/m^3^ increase in the concentrations of PM_2.5_ and PM_10_, the hospitalization risk of childhood acute bronchitis increased by 1.23% (95% CI: 0.21%–2.26%) and 1.33% (95% CI: 0.62%–2.05%), respectively (25). Another time-series analysis carried out in seven cities in Guangxi also confirmed that both PM_2.5_ and PM_10_ were significantly associated with the hospitalization of children due to lower respiratory infections (28). However, our study did not find a significant association between particulate matter and the hospitalization of children with acute bronchitis, and this result is also supported by some other studies (17, 18). During the study period, the daily average concentrations of PM_2.5_ and PM_10_ in Shantou were 28.1 μg/m^3^ and 47.6 μg/m^3^, respectively, which were much lower than the particulate matter concentrations in inland cities. This may be an important reason for the differences in the aforementioned study results.

Shantou, a city with a subtropical maritime climate on the southeast coast of China, is adjacent to the South China Sea. It is dominated by northerly winds in winter and southerly or southeasterly winds in summer, showing significant monsoonal characteristics. The city has abundant annual rainfall and moderate air humidity. The continuous sea breeze can effectively dilute and remove particulate pollutants in the air, reducing the actual exposure concentrations and the intensity of irritation to the human respiratory tract. At the same time, the thermal regulation effect of the ocean ensures that Shantou has mild winters and avoids extreme heat in summer, preventing the synergistic pathogenic effects of extreme temperatures and particulate pollution observed in inland cities. This may be an important climatic factor leading to the differences in research findings between this study and those conducted in inland megacities such as Guangzhou. The absence of significant adverse effects of particulate matter in this study may also be related to the different sources of aerosols in Shantou. The aerosols in Shantou originate not only from anthropogenic sources such as industry and traffic, but also from a large number of sea-salt particles generated by the evaporation of sea foam. Their chemical composition (such as sulfates and chloride ions) differs significantly from that of aerosols in inland cities, which are mainly of crustal and industrial origin. The toxicity of particles with different sources and chemical compositions varies considerably, and the marine components in coastal aerosols may reduce the respiratory pathogenic activity of PM₂.₅ and PM₁₀, thereby resulting in no significant association with hospitalizations for childhood acute bronchitis (29, 30).

Meanwhile, the results also indicated that the hospitalization of children with acute bronchitis in Shantou was only associated with two air pollutants, namely SO₂ and NO₂. For every 10 μg/m^3^ increase in the concentrations of SO₂ and NO_2_, the relative risk of hospitalizations for acute bronchitis in children was 1.2485 (95% CI: 1.1116–1.4023) and 1.1815 (95% CI: 1.1082–1.2596), respectively. This conclusion is consistent with most existing published studies. For instance, a time-series study covering nine cities in Sichuan Province, China, showed (25) a significant positive correlation between air pollutants and the hospitalization of children with acute bronchitis. Specifically, for every 10 μg/m^3^ increase in the concentrations of SO₂ and NO₂, the hospitalization risk of childhood acute bronchitis increased by 23.52% (95% CI: 11.52%–36.81%) and 12.47% (95% CI: 8.46%–16.64%), respectively. A study in Hefei (11), China, found that for each interquartile range (IQR) increase in NO₂ concentration, the hospitalization risk of childhood acute bronchitis rose by 3% (95% CI: 1%–5%). Another time-series study in Shanghai, China, also demonstrated (31) that for every 10 μg/m^3^ increase in the concentrations of SO₂ and NO₂, the hospitalization risk of childhood acute bronchitis increased by 11.12% (95% CI: 10.76%–11.48%), respectively. Relevant research in Hong Kong similarly confirmed a significant correlation between SO₂ and the hospitalization risk of children with bronchitis (12). A study using data on pediatric respiratory hospital admissions from five cities in Australia and two cities in New Zealand showed that hospital admissions for acute bronchitis in children were associated with SO₂ and NO₂ in each of these cities (32). However, a study in the United States did not find an association between the hourly peak of SO₂ and the emergency department visits for childhood bronchitis (33), which suggests that the association between air pollutants and the incidence of childhood respiratory diseases may be influenced by factors such as region.

Future studies should further expand the sample size and research scope, integrate environmental disparities between coastal and inland cities, and conduct in-depth exploration of the core mechanisms driving regional variations in the associations of particulate matter, SO₂, NO₂, and other pollutants with childhood acute bronchitis.

Results of the gender-stratified analysis in this study indicated that ambient air pollution in Shantou had a more pronounced impact on hospital admissions for acute bronchitis in boys than in girls, which was consistent with the findings of several studies conducted in other cities in China (27 28). Some studies found that both boys and girls were susceptible to ambient air pollutants, yet no significant gender-based differences were observed between the two groups (11, 25). Conversely, entirely opposite conclusions have also been reported; for instance, a time-series analysis in Shanghai revealed that females faced a higher risk of bronchitis induced by ambient air pollutants compared with males (31). This discrepancy may be attributed to inherent differences in physical structures and disparate environmental exposures between genders. In comparison with males, females have narrower respiratory tracts, where air pollutants tend to linger and accumulate, thereby hindering the timely clearance of pollutants by cilia-ted respiratory cells, triggering hyper reactive airway responses and the production of inflammatory cytokines, and ultimately inducing the onset or exacerbation of bronchitis (34). To date, there is no consistent explanation for the gender-based differences in susceptibility to ambient air pollutants, warranting further in-depth research. In the age-stratified analysis, we found that children aged 0–2 years were more vulnerable to the adverse effects of ambient air pollutants, which was in line with the results of a multi-city study conducted in Southwest China (25). Ages 0–2 are a critical stage for infants to rapidly establish innate and adaptive immunity, although the immune system is not yet fully mature at this stage. The airway epithelial barrier is not fully developed, and the capacities for antioxidation and inflammation regulation are relatively weak, making infants more prone to oxidative stress and excessive inflammatory responses when exposed to air pollutants, thereby triggering acute bronchial inflammation (35, 36). In addition, the lung tissue of children at this age is still in the stage of structural differentiation, with the number of alveoli, airway diameter, and lung ventilation function not yet fully developed. These children have a faster respiratory rate and a higher ventilation volume per unit of body weight, resulting in a relatively greater inhalation of pollutants over the same period. At the same time, their narrow airways mean that even mild inflammation can lead to significant airway obstruction, making clinical symptoms more pronounced and increasing the likelihood of hospitalization due to acute bronchial inflammation (2, 37, 38).

The seasonal stratification results of this study show that the impact of SO_2_ and NO_2_ on hospital admissions for acute bronchitis in children is significantly greater in the cold season than in the warm season. This is consistent with the conclusions reported in previous studies. For example, research in Guangzhou (27) and Guangxi (28), China, indicates that the impact of atmospheric pollutants on outpatient visits for bronchitis is more significant in the cold season, and a time series analysis in Hefei (11) also shows that the harmful effects of air pollutants on bronchitis patients are greater in the cold season. The seasonal differences in the impact of air pollutants on respiratory diseases may be related to factors such as the climate type, topography and meteorological conditions of each city. In addition, due to cold weather and large daily temperature differences, low temperatures can affect the body’s temperature regulation mechanisms, and cold air can stimulate the respiratory mucosa, weakening the body’s respiratory immunity and triggering a series of respiratory symptoms such as cough, sputum, and wheezing (39).

This study, from the perspective of Shantou, a coastal city in southern China, investigated the association between air pollutants and hospitalization for acute bronchitis in children, and identified the susceptible populations. Most previously published studies focused on the relationship between air pollution and various respiratory diseases, whereas the results of this study enrich the domestic epidemiological evidence regarding the association between air pollutants and acute bronchitis in children.

There were several limitations that cannot be ignored. Firstly, the hospitalization data for children with acute bronchitis in this study were only obtained from three major medical institutions in Shantou, failing to cover all hospitals in the city, which introduces a certain selection bias. Secondly, the air pollutant data in this study were obtained from fixed monitoring stations, and there is a certain discrepancy between the pollutant exposure levels at the monitoring stations and individual actual exposure levels. Using data from fixed stations to represent individual exposure may lead to a certain degree of exposure misclassification, thereby underestimating the health risks of air pollutants on acute bronchitis. Thirdly, hospitalizations for acute bronchitis may be influenced by individual characteristics such as lifestyle, behavioral patterns, immune system function, and genetic factors. This study did not include such information, so it is impossible to rule out the impact of these confounding factors on the results. Fourthly, this study is an ecological study conducted at the population level, which uses the overall levels of air pollutants to estimate individual exposure levels. The results may underestimate the actual impact of air pollutants on bronchitis, lead to ecological fallacy, limit the ability to infer causality, and cannot be extrapolated to the individual level. Finally, due to data availability limitations, this study only selected Shantou, a single coastal city in China. In the future, more data from additional coastal cities should be included for relevant analysis.

## Conclusion

In summary, our results indicated that short-term exposure to gaseous air pollutants (SO_2_ and NO_2_) was significantly associated with daily hospitalizations for acute bronchitis in children in Shantou, a coastal city in China. The effects were more pronounced in the boys, 0–2 years group and during the cold season. Our study adds epidemiological evidence of the association between air pollutants in Chinese coastal cities and hospital admissions for acute bronchitis in children, and also provides a reference for health authorities to formulate targeted air pollution intervention measures.

## Data Availability

The original contributions presented in the study are included in the article/Supplementary material, further inquiries can be directed to the corresponding author.

## Footnotes

1
https://www.aqistudy.cn/

2
http://www.escience.gov.cn
