# Supplementary material for: The impacts of air pollution on daily hospitalizations for acute bronchitis in children: a perspective from the coastal city of Shantou, China
Source: Front Public Health. 2026 May 8;14:1796373. doi: 10.3389/fpubh.2026.1796373 (PMC13194485; doi:10.3389/fpubh.2026.1796373)
Supplement: Supplementary file 1 [file Data_Sheet_1.DOCX]

The impacts of air pollution on hospital admissions for acute bronchitis in children: A Perspective from the coastal city of Shantou, China

* Corresponding author: Junduo Chen

E-mail: [chenjunduo1989@126.com](mailto:chenjunduo1989@126.com)

**Table S1** Spearman's correlation coefficients between meteorological factors and air pollutants in Shantou, China, 2015-2019. **P*<0.05, ***P*<0.01, ****P*<0.001.

**Table S2** Relative risk of daily hospitalizations for acute bronchitis in children associated with a 10 μg/m^3^ increase in SO_2_ and NO_2_ concentrations in the boys group in single-pollutant models

**Table S3** Relative risk of daily hospitalizations for acute bronchitis in children associated with a 10 μg/m^3^ increase in SO_2_ and NO_2_ concentrations in the girls group in single-pollutant models

**Table S4** Relative risk of daily hospitalizations for acute bronchitis in children associated with a 10 μg/m^3^ increase in SO_2_ and NO_2_ concentrations in the 0-2 years old group in single-pollutant models

**Table S5** Relative risk of daily hospitalizations for acute bronchitis in children associated with a 10 μg/m^3^ increase in SO_2_ and NO_2_ concentrations in the 3-6 years old group in single-pollutant models

**Table S6** Relative risk of daily hospitalizations for acute bronchitis in children associated with a 10 μg/m^3^ increase in SO_2_ and NO_2_ concentrations in the 7-14 years old group in single-pollutant models

**Table S7** Relative risk of daily hospitalizations for acute bronchitis in children associated with a 10 μg/m^3^ increase in SO_2_ and NO_2_ concentrations in the warm season in single-pollutant models

**Table S8** Relative risk of daily hospitalizations for acute bronchitis in children associated with a 10 μg/m^3^ increase in SO_2_ and NO_2_ concentrations in the cold season in single-pollutant models

**Table S9** The results of varying the degrees of freedom (6-9 *dfs*) for time trend

**Table S10** The results of varying the degrees of freedom (3-5 *dfs*) for mean temperature and relative humidity

**Table S1** Spearman's correlation coefficients between meteorological factors and air pollutants in Shantou, China, 2015-2019. **P*<0.05, ***P*<0.01, ****P*<0.001.

|  | PM_2.5_ | PM_10_ | SO_2_ | NO_2_ | O_3_ | Mean temperature | Relative humidity |
| --- | --- | --- | --- | --- | --- | --- | --- |
| PM_2.5_ | - |  |  |  |  |  |  |
| PM_10_ | 0.94^***^ | - |  |  |  |  |  |
| SO_2_ | 0.61^***^ | 0.66^***^ | - |  |  |  |  |
| NO_2_ | 0.66^***^ | 0.62^***^ | 0.62^***^ | - |  |  |  |
| O_3_ | 0.53^***^ | 0.58^***^ | 0.29^***^ | 0.20^***^ | - |  |  |
| Mean temperature | -0.39^***^ | -0.32^***^ | -0.33^***^ | -0.64^***^ | -0.11^***^ | - |  |
| Relative humidity | -0.29^***^ | -0.43^***^ | -0.13^***^ | -0.11^***^ | -0.53^***^ | 0.083^***^ | - |

**Table S2** Relative risk of daily hospitalizations for acute bronchitis in children associated with a 10 μg/m^3^ increase in SO_2_ and NO_2_ concentrations in the boys group in single-pollutant models

| Lag | SO2 | NO2 |
| --- | --- | --- |
| 0 | 1.0921 (0.9810-1.2158) | 1.0869 (1.0283-1.1489)* |
| 1 | 1.1712 (1.0581-1.2964)* | 1.1296 (1.0699-1.1926)* |
| 2 | 1.1428 (1.0342-1.2629)* | 1.1166 (1.0588-1.1776)* |
| 3 | 1.1354 (1.0261-1.2564)* | 1.1022 (1.0449-1.1627)* |
| 4 | 1.1893 (1.0771-1.3131)* | 1.1207 (1.0632-1.1814)* |
| 5 | 1.0425 (0.9426-1.1529) | 1.0284 (0.9740-1.0858) |
| 6 | 0.9637 (0.8703-1.0670) | 1.0263 (0.9718-1.0837) |
| 7 | 1.0105 (0.9134-1.1178) | 1.0456 (0.9903-1.1040) |
| 01 | 1.1917 (1.0532-1.3485)* | 1.1537 (1.0814-1.2308)* |
| 02 | 1.2539 (1.0935-1.4378)* | 1.2082 (1.1232-1.2996)* |
| 03 | 1.3143 (1.1316-1.5265)* | 1.2529 (1.1564-1.3576)* |
| 04 | 1.4089 (1.1993-1.6550)* | 1.3080 (1.1996-1.4262)* |
| 05 | 1.4209 (1.1943-1.6904)* | 1.3103 (1.1939-1.4380)* |
| 06 | 1.3862 (1.1501-1.6709)* | 1.3163 (1.1919-1.4537)* |
| 07 | 1.3875 (1.1367-1.6936)* | 1.3372 (1.2035-1.4859)* |

**Note:** **P* < 0.05

**Table S3** Relative risk of daily hospitalizations for acute bronchitis in children associated with a 10 μg/m^3^ increase in SO_2_ and NO_2_ concentrations in the girls group in single-pollutant models

| Lag | SO2 | NO2 |
| --- | --- | --- |
| 0 | 1.0341 (0.9564-1.1181) | 1.0344 (0.9900-1.0809) |
| 1 | 0.9982 (0.9254-1.0767) | 0.9860 (0.9429-1.0311) |
| 2 | 1.0245 (0.9512-1.1034) | 1.0131 (0.9699-1.0583) |
| 3 | 1.0799 (0.9998-1.1650) | 1.0283 (0.9850-1.0735) |
| 4 | 1.0673 (0.9910-1.1496) | 1.0039 (0.9608-1.0489) |
| 5 | 1.0617 (0.9864-1.1427) | 1.0214 (0.9781-1.0666) |
| 6 | 1.0108 (0.9389-1.0883) | 1.0044 (0.9615-1.0492) |
| 7 | 1.0209 (0.9486-1.0988) | 1.0232 (0.9796-1.0688) |
| 01 | 1.0216 (0.9333-1.1183) | 1.0138 (0.9626-1.0678) |
| 02 | 1.0324 (0.9340-1.1412) | 1.0193 (0.9616-1.0804) |
| 03 | 1.0744 (0.9639-1.1976) | 1.0338 (0.9703-1.1016) |
| 04 | 1.1061 (0.9838-1.2436) | 1.0342 (0.9659-1.1074) |
| 05 | 1.1339 (0.9999-1.2854) | 1.0429 (0.9696-1.1218) |
| 06 | 1.1335 (0.9918-1.2954) | 1.0427 (0.9652-1.1264) |
| 07 | 1.1436 (0.9922-1.3180) | 1.0524 (0.9699-1.1419) |

**Note:** **P* < 0.05

**Table S4** Relative risk of daily hospitalizations for acute bronchitis in children associated with a 10 μg/m^3^ increase in SO_2_ and NO_2_ concentrations in the 0-2 years old group in single-pollutant models

| Lag | SO2 | NO2 |
| --- | --- | --- |
| 0 | 1.0621 (0.9529-1.1838) | 1.0893 (1.0293-1.1529)* |
| 1 | 1.1290 (1.0183-1.2518)* | 1.1221 (1.0610-1.1867)* |
| 2 | 1.0798 (0.9748-1.1961) | 1.1147 (1.0555-1.1772)* |
| 3 | 1.1087 (1.0010-1.2280)* | 1.0963 (1.0378-1.1580)* |
| 4 | 1.2168 (1.1018-1.3439)* | 1.1269 (1.0677-1.1893)* |
| 5 | 1.0744 (0.9714-1.1884) | 1.0452 (0.9889-1.1046) |
| 6 | 0.9921 (0.8957-1.0987) | 1.0442 (0.9879-1.1038) |
| 7 | 1.0290 (0.9297-1.1390) | 1.0728 (0.9998-1.1487) |
| 01 | 1.1381 (1.0042-1.2900)* | 1.1494 (1.0756-1.2282)* |
| 02 | 1.1667 (1.0149-1.3410)* | 1.2032 (1.1166-1.2966)* |
| 03 | 1.2148 (1.0435-1.4142)* | 1.2430 (1.1451-1.3494)* |
| 04 | 1.3284 (1.1284-1.5640)* | 1.3031 (1.1926-1.4237)* |
| 05 | 1.3636 (1.1440-1.6255)* | 1.3165 (1.1969-1.4480)* |
| 06 | 1.3503 (1.1182-1.6308)* | 1.3333 (1.2045-1.4759)* |
| 07 | 1.3667 (1.1173-1.6718)* | 1.3717 (1.2314-1.5281)* |

**Note:** **P* < 0.05

**Table S5** Relative risk of daily hospitalizations for acute bronchitis in children associated with a 10 μg/m^3^ increase in SO_2_ and NO_2_ concentrations in the 3-6 years old group in single-pollutant models

| Lag | SO2 | NO2 |
| --- | --- | --- |
| 0 | 1.1101 (0.9572-1.2873) | 1.0033 (0.9259-1.0872) |
| 1 | 1.0785 (0.9345-1.2446) | 1.0146 (0.9369-1.0987) |
| 2 | 1.2173 (1.0618-1.3954)* | 1.0268 (0.9499-1.1099) |
| 3 | 1.1403 (0.9909-1.3122) | 1.0484 (0.9702-1.1328) |
| 4 | 0.9417 (0.8143-1.0891) | 0.9848 (0.9098-1.0661) |
| 5 | 0.9488 (0.8216-1.0957) | 0.9558 (0.8823-1.0354) |
| 6 | 0.8833 (0.7637-1.0215) | 0.9335 (0.8611-1.0122) |
| 7 | 0.9201 (0.7967-1.0625) | 0.9333 (0.8605-1.0123) |
| 01 | 1.1337 (0.9547-1.3462) | 1.0124 (0.9213-1.1124) |
| 02 | 1.2482 (1.0339-1.5068)* | 1.0254 (0.9224-1.1399) |
| 03 | 1.3131 (1.0697-1.6118)* | 1.0531 (0.9380-1.1824) |
| 04 | 1.2473 (0.9990-1.5574) | 1.0386 (0.9170-1.1763) |
| 05 | 1.2050 (0.9492-1.5298) | 1.0149 (0.8887-1.1589) |
| 06 | 1.1315 (0.8770-1.4599) | 0.9836 (0.8544-1.1323) |
| 07 | 1.0833 (0.8267-1.4197) | 0.9548 (0.8230-1.1077) |

**Note:** **P* < 0.05

**Table S6** Relative risk of daily hospitalizations for acute bronchitis in children associated with a 10 μg/m^3^ increase in SO_2_ and NO_2_ concentrations in the 7-14 years old group in single-pollutant models

| Lag | SO2 | NO2 |
| --- | --- | --- |
| 0 | 1.0377 (0.9447-1.1401) | 1.0433 (0.9896-1.0998) |
| 1 | 1.0105 (0.9227-1.1068) | 0.9864 (0.9347-1.0410) |
| 2 | 1.0298 (0.9420-1.1258) | 1.0104 (0.9586-1.0648) |
| 3 | 1.0864 (0.9949-1.1863) | 1.0282 (0.9763-1.0828) |
| 4 | 1.0764 (0.9846-1.1768) | 1.0049 (0.9532-1.0594) |
| 5 | 1.0627 (0.9728-1.1609) | 1.0233 (0.9713-1.0780) |
| 6 | 1.0222 (0.9354-1.1169) | 1.0059 (0.9544-1.0602) |
| 7 | 1.0342 (0.9470-1.1294) | 1.0194 (0.9673-1.0744) |
| 01 | 1.0329 (0.9266-1.1515) | 1.0202 (0.9585-1.0858) |
| 02 | 1.0444 (0.9261-1.1779) | 1.0226 (0.9534-1.0969) |
| 03 | 1.0893 (0.9562-1.2409) | 1.0369 (0.9607-1.1192) |
| 04 | 1.1249 (0.9772-1.2949) | 1.0375 (0.9556-1.1264) |
| 05 | 1.1528 (0.9915-1.3404) | 1.0469 (0.9590-1.1430) |
| 06 | 1.1580 (0.9861-1.3598) | 1.0473(0.9543-1.1493) |
| 07 | 1.1750 (0.9905-1.3939) | 1.0552 (0.9563-1.1642) |

**Note:** **P* < 0.05

**Table S7** Relative risk of daily hospitalizations for acute bronchitis in children associated with a 10 μg/m^3^ increase in SO_2_ and NO_2_ concentrations in the warm season in single-pollutant models

| Lag | SO2 | NO2 |
| --- | --- | --- |
| 0 | 0.9900 (0.8390-1.1682) | 0.9635 (0.8574-1.0828) |
| 1 | 1.0342 (0.8840-1.2101) | 1.0166 (0.9072-1.1391) |
| 2 | 1.1508 (0.9887-1.3394) | 1.1031 (0.9886-1.2309) |
| 3 | 1.0760 (0.9221-1.2555) | 0.9986 (0.8929-1.1167) |
| 4 | 1.0949 (0.9379-1.2782) | 1.0103 (0.9042-1.1288) |
| 5 | 1.1249 (0.9660-1.3100) | 1.0369 (0.9299-1.1563) |
| 6 | 1.0179 (0.8723-1.1879) | 1.0131 (0.9078-1.1306) |
| 7 | 1.0187 (0.8712-1.1913) | 1.0171 (0.9101-1.1368) |
| 01 | 1.0188 (0.8394-1.2366) | 0.9860 (0.8583-1.1326) |
| 02 | 1.1143 (0.8984-1.3821) | 1.0554 (0.9015-1.2355) |
| 03 | 1.1533(0.9092-1.4630) | 1.0498 (0.8817-1.2499) |
| 04 | 1.2096 (0.9311-1.5715) | 1.0531 (0.8715-1.2725) |
| 05 | 1.2933 (0.9725-1.7200) | 1.0734 (0.8760-1.3154) |
| 06 | 1.3050 (0.9593-1.7753) | 1.0808 (0.8685-1.3449) |
| 07 | 1.3218 (0.9494-1.8402) | 1.0928 (0.8631-1.3837) |

**Note:** **P* < 0.05

**Table S8** Relative risk of daily hospitalizations for acute bronchitis in children associated with a 10 μg/m^3^ increase in SO_2_ and NO_2_ concentrations in the cold season in single-pollutant models

| Lag | SO2 | NO2 |
| --- | --- | --- |
| 0 | 1.0693 (0.9615-1.1893) | 1.0435 (0.9917-1.0981) |
| 1 | 1.0975 (0.9901-1.2166) | 1.0424 (0.9903-1.0973) |
| 2 | 1.0345 (0.9334-1.1466) | 1.0298 (0.9794-1.0828) |
| 3 | 1.0867 (0.9795-1.2055) | 1.0691 (1.0169-1.1241)* |
| 4 | 1.0784 (0.9707-1.1980) | 1.0433 (0.9915-1.0978) |
| 5 | 0.9277 (0.8341-1.0317) | 0.9759 (0.9270-1.0274) |
| 6 | 0.8347 (0.7504-0.9283)* | 0.9538 (0.9059-1.0043) |
| 7 | 0.9133 (0.8222-1.0144) | 0.9818 (0.9323-1.0341) |
| 01 | 1.1283 (0.9934-1.2816) | 1.0645 (1.0003-1.1328)* |
| 02 | 1.1340 (0.9804-1.3117) | 1.0791 (1.0037-1.1602)* |
| 03 | 1.1848 (1.0081-1.3923)* | 1.1288 (1.0394-1.2259)* |
| 04 | 1.2280 (1.0284-1.4663)* | 1.1607 (1.0580-1.2733)* |
| 05 | 1.1727 (0.9662-1.4234) | 1.1410 (1.0306-1.2633)* |
| 06 | 1.0559 (0.8562-1.3024) | 1.1027 (0.9884-1.2303) |
| 07 | 1.0003 (0.7985-1.2531) | 1.0896 (0.9688-1.2254) |

**Note:** **P* < 0.05

**Table S9** The results of varying the degrees of freedom (6-9 *dfs*) for time trend

| Air pollutants | Time(*df*=6) | Time(*df*=7) | Time(*df*=8) | Time(*df*=9) |
| --- | --- | --- | --- | --- |
| SO_2_ | 1.4164 (1.2876-1.5580)* | 1.2561 (1.1343-1.3908)* | 1.2301 (1.1091-1.3643)* | 1.2301(1.1074-1.3662)* |
| NO_2_ | 1.2734(1.2007-1.3505)* | 1.1815 (1.1082-1.2596)* | 1.1361(1.0614-1.2160)* | 1.1475(1.0698-1.2310)* |

**Note.** SO_2_: lag05 day; NO_2_: lag07 day. **P* < 0.05.

**Table S10** The results of varying the degrees of freedom (3-5 *dfs*) for mean temperature and relative humidity

| Pollutants | Mean temperature | | |  | Relative humidity | | |
| --- | --- | --- | --- | --- | --- | --- | --- |
|  | *df*=3 | *df*=4 | *df*=5 |  | *df*=3 | *df*=4 | *df*=5 |
| SO_2_ | 1.2561 (1.1343-1.3908)^*^ | 1.2562 (1.1344-1.3909)^*^ | 1.2515 (1.1303-1.3857)* |  | 1.2561 (1.1343-1.3908)* | 1.2563 (1.1346-1.3911)* | 1.2564 (1.1346-1.3913)* |
| NO_2_ | 1.1815 (1.1082-1.2596)^*^ | 1.1820 (1.1087-1.2602)* | 1.1792 (1.1061-1.2572)* |  | 1.1815 (1.1082-1.2596)* | 1.1816 (1.1083-1.2597)* | 1.1818 (1.1085-1.2600)* |

**Note.** SO_2_: lag05 day; NO_2_: lag07 day. **P* < 0.05.
